# A META-COGNITIVE GROUP INTERVENTION FOR OLDER ADULTS WITH SCD OR MCI: RCT AND QUALITATIVE EVALUATION

**DOI:** 10.1093/geroni/igae098.0205

**Published:** 2024-12-31

**Authors:** Shlomit Rotenberg, Nicole Anderson, Deirdre Dawson

**Affiliations:** University of Toronto, Toronto, Ontario, Canada; Baycrest Academy for Research & Education, Toronto, Ontario, Canada; University of Toronto, Toronto, Ontario, Canada

## Abstract

We explored ASPIRE, a meta-cognitive group intervention aimed to improve engagement in daily activities of older adults with subjective cognitive decline (SCD) or mild cognitive impairment (MCI). We performed a double-blind randomized controlled trail, and complementary qualitative study. Participants were randomly assigned to ASPIRE (n=131) or a Brain Education control group (n=133). The primary quantitative outcome was improvement in performance of daily activities not trained in either intervention, using the Canadian Occupational Performance Measure (COPM). A linear mixed effects regression model found clinically significant improvement (change ≥ 2 points on the COPM) post intervention in 32.5% of untrained activities in ASPIRE; and 30.6% in Brain Education, with no significant group effects (exp(ß)=0.96, z=-0.15, p=.879). Improvements from pre- to post-intervention were found in both arms on secondary measures of subjective cognition and self-efficacy, with no significant group effects. Qualitative data were written responses about the experience of ASPIRE or Brain-Education collected post intervention, analysed using inductive thematic content analysis. ASPIRE participants were pleased with the Group Context, that included learning from, and helping others with shared experiences of cognitive concerns. They described ASPIRE as applicable to Everyday Life, providing practical strategies and motivating action through accountability to the group. Brain-Education participants were pleased with the Content, specifically the topic areas, described as interesting and stimulating. Both groups were pleased with the facilitation approach. We conclude that ASPIRE and Brain-Education have similar potential to improve subjective cognition, self-efficacy, and engagement in daily activities, but the mechanisms by which they work are different.

